# Data-Driven Modeling and Rendering of Force Responses from Elastic Tool Deformation

**DOI:** 10.3390/s18010237

**Published:** 2018-01-15

**Authors:** Arsen Abdulali, Ruslan Rakhmatov, Tatyana Ogay, Seokhee Jeon

**Affiliations:** Department of Computer Engineering, Kyung Hee University, Yongin-si 446-701, Korea; abdulali@khu.ac.kr (A.A.); rakhmatovruslan@khu.ac.kr (R.R.); ta.ogay92@gmail.com (T.O.)

**Keywords:** haptic rendering, tool deformation, data-driven modeling, force feedback

## Abstract

This article presents a new data-driven model design for rendering force responses from elastic tool deformation. The new design incorporates a six-dimensional input describing the initial position of the contact, as well as the state of the tool deformation. The input-output relationship of the model was represented by a radial basis functions network, which was optimized based on training data collected from real tool-surface contact. Since the input space of the model is represented in the local coordinate system of a tool, the model is independent of recording and rendering devices and can be easily deployed to an existing simulator. The model also supports complex interactions, such as self and multi-contact collisions. In order to assess the proposed data-driven model, we built a custom data acquisition setup and developed a proof-of-concept rendering simulator. The simulator was evaluated through numerical and psychophysical experiments with four different real tools. The numerical evaluation demonstrated the perceptual soundness of the proposed model, meanwhile the user study revealed the force feedback of the proposed simulator to be realistic.

## 1. Introduction

Surgical practice requires both theoretical background and operative experience. The experience of the surgical operation is obtained during hundreds of extensive trials of training [1]. A virtual reality-based simulation is a modern approach to the development of surgical skills. The crucial part of such simulations is an accurate haptic feedback, e.g., force [2], torque [3], and vibrotactile [4] responses. In such medical and haptic-enabled simulators, the physical interaction with a virtual human tissue or organ usually happens through the medium of interaction - haptic device. The interaction tool of the haptic device replaces a virtual medical instrument, and all collision responses are physically recreated and provided to the user. The process of computing and displaying such responses is referred to as haptic rendering.

One of the actively researched areas in haptic-enabled medical simulator is the haptic rendering of soft and deformable matters, e.g., soft tissue [5], organs [6], and even soft tools [7] used in medical procedures. In general, haptic rendering algorithms for deformable objects estimate the feedback stimuli relying upon the computational model that mimics the real physical process of the tool-deformable object collision. Balancing the degree of complexity of this computational model is sometimes a critical issue for a successful application of the haptics technology. Too crude model cannot guarantee the simulation error low enough to be perceptually negligible, while too complex model cannot ensure 1 kHz update rate of haptic simulation. Thus, the haptic model and rendering algorithm are designed in accordance with the trade-off.

There is a branch of works that lies at one extreme side in this trade-off: simple parametric models based simulation. In this approach, the input-output relationship is described by simple polynomial equations, e.g., the stiffness of simple geometric objects approximated by penalty [8] or constraint-based [9] methods based on the Hook’s law. The idea of constraint-based algorithm can be extended for non-linear stiffness rendering by employing second-order polynomial, e.g., Fortmeier et al. proposed to model the pre-puncture phase of the needle insertion using a non-linear spring model in liver puncture simulation [10]. At the other extreme of the trade-off, models based on continuum mechanics are used. The solution for the global deformation is approximated by applying discretization methods, e.g., mass-spring system [11], finite element method (FEM) [12], etc. However, these techniques require preliminary knowledge about the object structure and physical properties of the object material. In general, due to computational complexity of continuum mechanics-based models, a real-time rendering performance is impractical.

The compromise solution for object deformation modeling in terms of computational complexity and rendering quality is a data-driven approach. The data-driven approach is an interpolation algorithm that blindly maps the applied input of the contact to the feedback stimuli, omitting the meaning of underlying physics of the deformation. The input-output relation of this approach is provided by the computational model, which is trained beforehand the rendering. Training data are collected during a real physical tool-object contact with the help of sophisticated sensors. Thus, the data-driven model tends to produce similar feedback to the one during the real interaction.

Recently, a data-driven modeling approach has been successful in haptic simulations of the contact dynamics between a deformable target object and a rigid tool (see Section 2.2 for complete review). Considering that in many surgical cases, the tool, as well as contacting tissue deforms and produces significant haptic feedback, force responses due to tool deformation should be one of the next target to be considered. However, to the best of our knowledge, a data-driven approach was not utilized in simulations of a tool deformation yet. Furthermore, it is difficult to directly apply an available data-driven algorithm since in most cases, input space of the model is represented in the absolute coordinate frame of the recording device, which cannot represent a moving tool. There are few approaches for the tool deformation based on simplified continuum models (see Section 2.1 for in depth review), but such simplifications restrict a degree of freedom of the deformation making the haptic feedback less accurate, and their continuum-model nature makes parameter tuning very challenging to achieve realistic haptic feedback.

In this article, we present a novel data-driven model that approximates force responses from elastic deformation of inhomogeneous tools. Our input space design consists of a six dimensional input vector that describes the initial contact point on the tool’s surface and the state of the tool deformation. Since the state of deformation is represented by a translation vector of the initial contact point, our novel approach supports complex interactions such as self and multi-contact collisions. In order to utilize the proposed model in a real-time simulation, we have designed a rendering pipeline where a virtual tool was represented by collision and haptic models. This pipeline allows our algorithm to work with any available collision detection algorithms and to be run independently from visual simulation, making our algorithm deployable to existing systems with minimal modifications. We summarized a list of our major contributions as follows:

### Contributions

- A data-driven model for elastic tool deformation was designed. Assuming that a virtual deformable tool interacts only with a flat surface, we designed the model that approximates force responses at the tool’s origin based on six-dimensional input vector, which describes a contact position and state of tool deformation in the local coordinate system of the tool (see Section 4.1).
- A data acquisition setup that captures the input-output data-points from real tool-surface interaction was built. In order to sense a position and orientation of the tool and force responses at the tool’s origin, we customized the standard gimbal encoder of a haptic device. The initial position of the tool-surface contact was detected by the capacitive touchscreen.
- A proof-of-concept rendering application was developed. The rendering quality of the simulator was assessed by modeling and rendering four different tools through numerical and psychophysical experiments.

The remainder of the article is structured as follows. The background knowledge and taxonomy of object and tool deformation rendering are provided in Section 2. The model design and discussion about basic benefits and limitations are provided in Section 3. In Section 4, we developed a proof-of-concept rendering simulator. In order to evaluate our simulator, we performed numerical and psychophysical evaluations in Section 5 and Section 6 respectively. The conclusion and future work are discussed in Section 7.

## 2. Background and Related Works

The research on the general haptic rendering of virtual objects’ geometry begun with simple linear models where it was assumed that all target objects in a virtual scene were isotropic and homogeneous, and the interaction with them was limited to a single point. For example, Thomas et al. developed a haptic rendering device that allows users to interact with rigid primitives, such as cube and sphere through haptic interface point (HIP), i.e., the end point of the haptic device [13]. This idea evolved in constraint-based methods where the force response is computed based on the penetration depth of the HIP [9,14]. It was assumed that haptic feedback is homogeneous for the whole object and linearly related to the penetration depth. Recently, Fortmeier et al. proposed a multiproxy approach that approximates normal and tangential forces based on computed tomography (CT) images [15]. Kaluschke et al.computed interaction forces between mesh object and streaming point clouds based on interpenetration volume [16]. In [10,17], the authors developed and evaluated a visuo-haptic framework that employs the direct volume rendering concept over the partially segmented CT images.

In order to render nonlinear force response from inhomogeneous objects, researchers utilized continuum models of the global geometry deformation. The numerical computation of global deformation is obtained with the help of discretization methods, such as mass-spring system and finite element method. Methods of continuum deformation is found to be useful for haptic rendering in many surgical simulators, e.g., tissue cutting [18,19], needle insertion [20], stitching [21], palpation [22], laparoscopic surgery [23].

### 2.1. Tool Deformation Rendering

One of the pioneering work in tool deformation rendering was proposed by Baxter et al. where the authors simulated the deformation of a three-dimensional brush while drawing on two-dimensional canvas [24]. Force responses are calculated based on a simple piecewise linear function of the brush head penetration. In [7], the authors proposed surgical simulator of inserting a catheter into a cystic duct. In their setup, two virtual forceps were used for holding a virtual duct and catheter. They applied a hybrid system of mass-spring and FEM models where the object (duct) was modeled by FEM model, and the tool (catheter) was represented by the mass-spring system. Gockel et al. introduced a system for simulation of the tooth cleaning process [25]. They modeled each bristle of the brush using FEM approach. Due to the time complexity, they were able to model only one bristle at frequency of 1 kHz. In order to render a complete brush, they replaced a haptics device by a computer keyboard.

Laycock et al. presented another method for tool deformations [26]. The authors employed the finite element method for tool deformation during interaction with convex objects. This work was extended in [27,28] by applying a hybrid collision detection algorithm that allows interaction with non-convex objects. The shape of the tool was simplified to a cylindrical rod that was discretized into several volumetric elements (beams) having two endpoints (nodes). The global deformation of the tool was described by position and orientation of each node. Garre et al. proposed the approach of human hand modeling where each finger of the hand was approximated by a linear co-rotational finite element model [29] and coupled with a rigid palm using stiff damped springs [30]. The skeletal constraints of the hand were additionally included in [31]. However, the hand model was still limited to linear elasticity and the hand was represented as a homogeneous object.

Summarizing the related research on tool deformation modeling, we can ascertain several common characteristics. In order to keep the haptic update rate at least 1 kHz, the authors resorted to simplifications over the tool geometry or over discretization methods of the continuum deformation models. Since a shape of the tool was changed continuously during rendering, extra computation was required for collision detection. In our approach, we omit the computation of the global deformation of the tool geometry by applying a data-driven formulation.

### 2.2. Data-driven Approach

The research on the data-driven haptic rendering started from fitting piecewise linear models, e.g., MacLean et al. estimated stiffness, damping, and inertia parameters from recorded force signals [32]. In [33], the authors recorded cutting forces from surgical scissors and rendered them using piecewise linear models. Hover et al. proposed two offline interpolation schemes for the rendering of one-dimensional deformation of visco-elastic objects where they increased the density of the force field beforehand the simulation [34]. Sparse recordings of the real interaction were interpolated using simplex-based piecewise linear approximation and radial basis functions. This work was extended to the rendering of viscous fluids in [35], and slipping phenomena in [36]. In order to improve modeling performance, Abdelrahman et al. proposed to reduce a training dataset by segmenting the shape of the mesh model into flat regions [37]. Assuming that the object is isotropic and homogeneous, each region can be described by a single contact. This idea was extended in [38] to incorporate inhomogeneity of the object material where the object shape was partitioned by implying a force response clustering. Fong et al. proposed an approach for modeling elastic object deformation during sliding contact [39]. Recently, this work was extended to the modeling of visco-elastics effects during the sliding contact in [40]. A common characteristic of the most data-driven techniques is that they focused on rendering force responses from static objects where the tool was assumed to be rigid. In our research, we aim to build a data-driven model for rendering force responses from elastic tool deformation.

## 3. Tool Deformation Modeling

In general, a data-driven model design incorporates two main steps. First, the input and output spaces of the model are defined. Model input is an *n*-dimensional vector representing the state of the deformation, whereas the output is an *m*-dimensional response stimuli. The dimension of the input space denotes the degree of freedom of the model. By increasing the number input variables, the model captures more features that determine the deformation of the target object. Consequently, the feedback output of the model becomes more accurate. However, since the amount of data required for training increases exponentially with each additional input variable, it becomes challenging to cover the whole input space during data collection. This results in the dilemma whether to improve the model quality by an additional input variable or to make the system less complicated and more practical. The second important aspect in data-driven model design is a formulation of the mathematical model that learns input-output pairs and guarantees stable interpolation during rendering while meeting the demand for the 1 kHz update rate.

The rest of this section covers the model design by defining the input and output spaces and explains the mathematical model that stores and interpolates input-output pairs. At the end of the section, we will discuss model benefits, limitations, and future directions.

### 3.1. Assumptions

A tool-object interaction is complex phenomena where a wide range of haptic properties of both tool and object must be taken into account. Furthermore, a grip and internal dynamics of the hand should be considered. Considering that it is infeasible to cover all aspects of interaction in a single research, we imposed several assumptions. Since a haptic model of the tool deformation is the focus of the current article, we assume that the interacting object is a flat rigid surface. In addition, in order to avoid modeling of friction and stickiness of the plane, we assume that no sliding occurs during the contact. In our future work, we plan to resolve these assumptions gradually.

### 3.2. Model Input and Output

**Figure 1:**
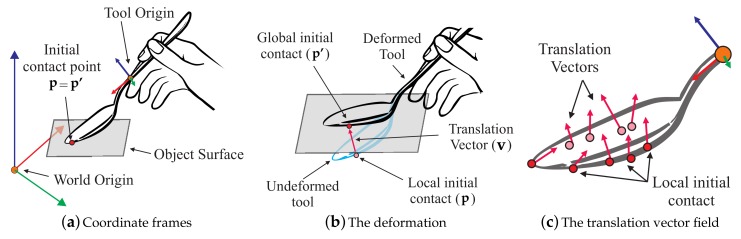
Descriptions of the model input space: (**a**) the input space is defined with respect to the origin of a tool; (**b**) a six-dimensional input vector consists of a position of an initial contact **p** and a translation vector, **v** that describes the state of deformation; (**c**) A set of recorded input vectors are used in interpolation, and the force response is approximated at the tool’s origin during rendering.

We propose a data-driven model with a six-dimensional input space design. First three dimensions describe the position of the initial contact in tool’s local coordinate frame, which is denoted as the *local initial contact* (see **p** in Figure 1a). **p** is determined at the moment of initial contact and remains constant in the local coordinate frame during a contact. The last three dimensions are related to the position of the initial contact that remains constant in the global coordinate frame, i.e., initial contact point on the object surface (see **p’** in Figure 1b). We denote this as *global initial contact*. At the initial moment of the contact, both the local and global initial contact points represent the same point (see Figure 1a). However, when the tool begins deforming, **p** penetrates into the surface and moves away from **p’** as illustrated in Figure 1b. The difference between the two point can explain the state of deformation, and the difference vector **v** = **p’** − **p** is referred to as *translation vector*. The translation vector becomes the last three input dimensions.

The resultant input vector of the model is $u=\langle p,v\rangle$. Taking into account that interaction happens in three-dimensional space, the final input space of the model can be expressed in six dimensions $u=\langle p_{x},p_{y},p_{z},v_{x},v_{y},v_{z}\rangle$.

It is important to notice that translation vector **v** differs from deformation vector used in the continuum mechanics (a vector representing the total movement of a particle on a deformed surface). In general, calculating this deformation vector needs the actual geometry of the deformed surface, which requires geometry recalculation of the tool deformation. This information is computationally expensive and is not generally available in a data-driven modeling scenario. Thus, we decided to avoid it. Instead, we utilize a vector representing a change of the initial contact point during deformation.

The model output is a three-dimensional force vector f at the tool’s origin (see Figure 1a). The force response under a certain deformation should be explicitly determined by an initial contact point and the current position of the external force application, i.e., the encountered surface in our case. Thus, the initial contact point and the translation vector can fully explain the response force at the tool’s origin. In our implementation, both of the inputs are presented in the local coordinate frame of the tool.

### 3.3. Mathematical Model

We develop a computational formulation that relates aforementioned input-output spaces. Data-driven model of the tool deformation can be understood as a function, whose parameters are optimized based on given observations, i.e., a set of input-output recordings from the real tool-surface interaction. The process of computing model parameters is referred to as *modeling*. During the simulation, the sequence of input vectors is fed into the resultant model while the model computes a continuous output of the force feedback.

In literature, there were several approaches proposed for input-output mapping. The most straightforward way is to utilize simplex-based methods [34] where the data are stored in a look-up table and approximated using weighted interpolation. The second way is to utilize feed-forward neural networks [41] that continuously compute a feedback output based on a given input during rendering. In this work, we adopted a radial basis functions network (RBFN), since it was successfully used in the majority of data-driven simulators of the object deformation [35,39,40].

An RBFN consists of three layers, i.e., input, hidden, and the output layer. The nodes of the hidden layer represent non-linear RBF activation functions. The input vector belongs to an *n*-dimensional Euclidean vector space, $u\in R^{n}$, and is transformed to a single scalar value of the output layer, $\phi :R^{n}\to R$, which can be described by

(1) $$f_{t}\left(u\right)=\sum_{j=1}^{N}w_{tj}\phi (∥u-q_{j}∥)+\sum_{k=1}^{L}d_{tk}g_{k}\left(u\right)\;\;\;\;u\in R^{n},$$

where $w_{tj}$ is the weight constant, $q_{j}$ is the center of the radial basis function, the function $g_{k}\left(u\right)$ (k=1,…,L) forms a polynomial term, *t* is basis of the output space of the model, and ϕ(∥·∥) is a radial basis activation function. Since cubic spline $\phi \left(r\right)=r^{3}$ is chosen as the RBF kernel, the polynomial term is needed to ensure stability [42].

The weight constants $w_{t}$ and polynomial coefficients $d_{t}$ can be estimated by solving the following linear system:

(2) $$\left(\begin{array}{cc} \Phi & G\\ G^{T} & 0 \end{array}\right)\left(\begin{array}{c} w_{t}\\ d_{t} \end{array}\right)=\left(\begin{array}{c} f_{t}\\ 0 \end{array}\right),$$

where $\Phi_{ij}=\phi (∥u_{i}-u_{j}∥)$, and $G_{ik}=g_{k}\left(u_{i}\right)$ for i,j={1,…,N}, and k={1,…,L}, respectively. Since the RBFN provides only vector-scalar mapping, each basis of the force vector $f_{t}$ is computed independently.

### 3.4. Benefits and Limitations

As long as the input space of the model is represented in the local coordinate system of the tool, our approach is independent of the recording and rendering devices. In addition, the model requires the only position of the initial contact where the translation vector is computed based on position and orientation of the tool. This makes our model compatible with existent collision detection algorithms.

**Figure 2:**
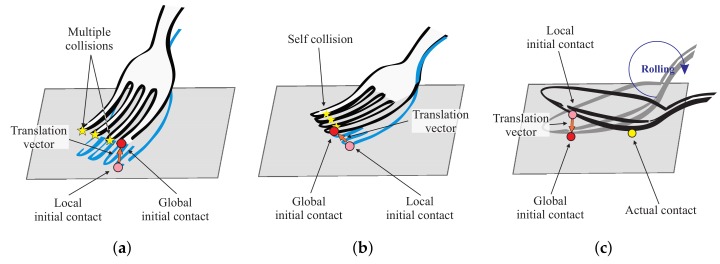
Complex contact deformation: our data-driven model provides a non-linear input-output mapping that allows simulating the following deformations (**a**) multiple-contact; (**b**) self-collision; and (**c**) rolling-contact.

The model is able to describe complex interactions, such as self-collision, multiple and rolling contacts (see Figure 2), as it provides input-output mapping regardless of the deformation complexity and output force nonlinearity. In particular, rolling of the tool invokes the changes in the direction of the translation vector, providing unique input vectors. Even in the extreme case when the rolling happens with negligible tool deformation, there is always unique translation vector pointing outwards of the tool’s shape (see Figure 2c). Another benefit of the framework is that the model can be easily extended. For example, by adding three-dimensional velocity-dependent values to the model input, it can be easily extended to incorporate visco-elastic behavior of a tool.

## 4. Tool Deformation Simulator

Data-driven paradigm is a three-step process, i.e., data collection, modeling, and rendering. This section describes the three steps that we have adapted in our tool deformation simulator.

**Figure 3:**
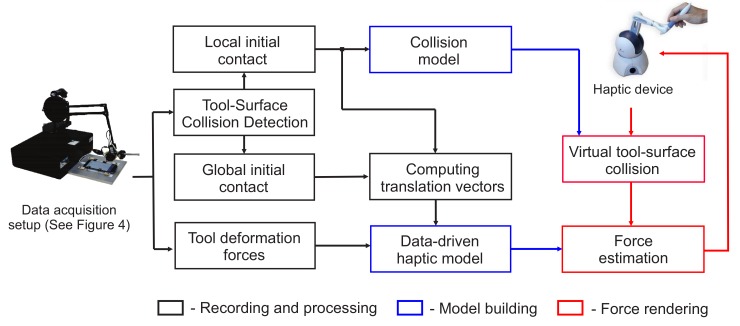
System overview.

Our simulator utilizes the data-driven model from Section 3. The pipeline of our simulator is depicted in Figure 3. In accordance with the input space of the model, we have designed recording setup (see Section 4.1) that captures input-output pairs during the real interaction. In Section 4.2, a representative set of input-output pairs is selected. Finally, we build *Collision* and *Haptic models* which are used in rendering application (see Section 4.3).

### 4.1. Data Acquisition and Preprocessing

**Figure 4:**
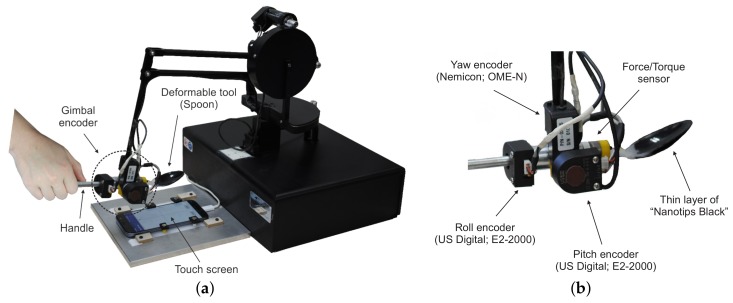
Data recording setup. A user holds the handle of the device and presses the tool to surface of the touchscreen. Data from the tool deformation is recorded when the tool is in contact with the touchscreen. (**a**) Recording hardware; (**b**) Gimbal encoder.

We designed and assembled a manual recording setup that captures data from three sources (see Figure 4a). Three-dimensional force signal was captured by force/torque sensor (Nano17; ATI Industrial Automation, Inc., Apex, NC, USA) using NI DAQ acquisition board (PCI-6220; National Instruments, Austin, TX, USA) with a sampling rate of 1000 Hz. The position and orientation of the tool’s origin were recorded by a haptic device (PHANToM Premium 1.5; Geomagic Inc., Rock Hill, SC, USA). In order to acquire the orientation of the tool, we design a custom gimbal encoder (see Figure 4). The pitch and roll angles were measured by incremental encoders with angular resolution 0.045° (E2-2000; US Digital, Vancouver, WA, USA). The yaw angle was measured by a standard incremental encoder (OME-N; Nemicon, Tokyo, Japan) with angular resolution 0.18°, which was mounted by the manufacturer of the haptic device. The raw data from the gimbal encoder was acquired through the original 24-pin Mini Delta Ribbon (MDR) interface of the haptic device using the library (OpenHaptics 3.4; 3D Systems, Inc., Rock Hill, SC, USA). In order to compute the position and orientation of the tool’s origin, we implemented the forward kinematics of the haptic device [43] considering the angular resolution of the custom gimbal encoder.

The collision point between the tool and a flat surface was recorded using a capacitive touch screen of the smartphone (Galaxy S7; Samsung Electronics Co. Ltd., Suwon, Korea). In order to make the tool sensitive for the touch screen, we coated it with a liquid that is comprised of evenly dispersed ultra-fine conductive nano-particles (Nanotips Black; Nanotips Inc., Vancouver, BC, Canada). The position of the initial contact was recorded from the smartphone through the network. The package delivery latency of the network was less than one millisecond. The position of the initial contact with respect to the world coordinate system (coordinate system of the haptic device) is stored and translated to the local coordinate system of the tool during the deformation. First translated initial contact point represents the *local initial contact* and each subsequent translated initial contact point represents the *global initial contact*. When the position and orientation of the tool’s origin are changed, the global initial contact point moves away from the local initial contact. The vector pointing from the local to global initial contact points is a *translation vector*. The input vector of the proposed model u is a combination of the local initial contact and the translation vector.

To minimize the noise, force signals were filtered using a three pole Butterworth low-pass filter with a cut-off frequency 25 Hz. The cut-off frequency of the filter was selected accordingly to the human hand movement capability [44]. Similarly, the position data was smoothed using a third-order Butterworth low-pass filter with a with a cut-off corner frequency 25 Hz. Only the data points where the tool was in touch with the object were considered while other redundant data points were removed.

**Figure 5:**
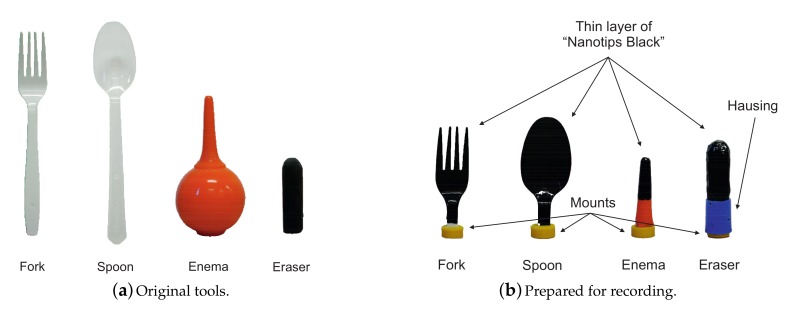
A set of real tools for evaluation: (**a**) illustration of original tools; and (**b**) modified versions of tools prepared for numerical and psychophysical experiments.

In order to evaluate the proposed simulator, we collected four deformable tools (see Figure 5a). Each tool was selected in accordance with characteristics of the tool that we want to evaluate. In order to evaluate multi-contact and self-collision interactions, we selected ‘Fork’. ‘Spoon’ was selected to assess non-linear force feedback of complex deformation with moderate rolling. ‘Enema’ shows the deformation with high compliance and relatively small forces. The ‘Eraser’ is made of stiff elastic rubber with a hemispherical tip that also allows considerable rolling during deformation.

Each tool was cut at the grip point, i.e., a point between index and thumb fingers while holding a tool. We refer to this point as tool’s origin. Then, a 3D-printed adapter was attached to the edge of the cut for mounting the tools to the recording setup as shown in Figure 5b. It is important to notice that the cut could cause mechanical changes in a tool’s structure. However, the major contribution to the haptic feedback during tool-object interaction is provided by the deforming part of a tool. The upper part of a tool, which is on the other side of the grip point, is grasped in person’s hand contributing negligible feedback.

In order to detect the contact with the touchscreen, each tool was coated with a thin layer of Nanotips Black (see Figure 5b). For the best performance, the coating layer was dried for 2 days. Hereafter, the modified versions of tool’s presented in Figure 5b are referred to as real tools. The set of real tools were used for numerical and psychophysical experiments (see Section 5 and Section 6).

### 4.2. Sample Selection

Due to the high sampling rate, sensors produce a massive amount of data during a recording session. The only small portion of the collected data contributes to the model significantly. The rest of the data are considered redundant. Furthermore, when overabundant samples are used in modeling, the risk of model over-fitting increases. Additionally, the processing time in model building and rendering also increases.

In the field of haptics, there are two main classes of the sample selection algorithms. First class is represented by wrapper methods where the complete set is trained and reduced iteratively [45]. The selection process of this algorithm is guided by the approximation model, which enhances the selection quality. However, for the large datasets, the processing time becomes everlasting. The second class of algorithms is based on hierarchical clustering, which recursively partitions the input space of the initial set and selects a single representative sample from each group of partitions [46,47].

Recently, we developed Stimuli-Magnitude-Adaptive Sample Selection (SMASS) algorithm that utilizes BSP-tree for hierarchical clustering of the input space in such a way that the ratio between the standard deviation and the mean for each output group is kept nearly constant below than a given threshold. The main characteristic of this algorithm is that it follows Weber’s law, which incorporates Just Noticeable Difference (JND) of the physical stimuli. JND is the minimum level of stimulation that a person can detect at least half the time. For example, subject can easily detect the difference between 0.7 N and 1 N force whereas the difference between 10.7 N and 11 N is unnoticeable. Thus, SMASS tends to select more samples where the force magnitude is lower.

In the present work, we decided to apply the SMASS algorithm, since we need to select the minimal number of samples while the relative error of the force responses remains below the JND level.

### 4.3. Model Estimation

Once the representative features are extracted, the desired weight vector $w_{t}$ and polynomial coefficients $d_{t}$ of the RBFN model can be calculated based on the inverse of the interpolations matrix, as follows

(3) $$\left(\begin{array}{c} w_{t}\\ d_{t} \end{array}\right)={\left(\begin{array}{cc} \Phi & G\\ G^{T} & 0 \end{array}\right)}^{-1}\left(\begin{array}{c} f_{t}\\ 0 \end{array}\right).$$

Since the size of the interpolation matrix is proportional to the square of the number of selected samples, it becomes computationally expensive to find the inverse of the interpolation matrix. This problem can be solved using the $l_{1}$ minimization technique as showed in [38]. There is a wide range of algorithms solving $l_{1}$ minimization routines, but based on the survey in [48], we found SpaRSA [49] as the most suitable. It is important to notice that $w_{t}$ and $d_{t}$ are computed for three basis of the force vector independently. In order to compute force responses during rendering (see Equation (3)), three matrices should be provided, i.e., *w*, *d*, and *q*. A set of these matrices is referred to as *Haptic Model*.

During model building, the input vector can be directly derived from the sensor readings, i.e., data from the touch-contact sensor and the PHANToM’s position encoder. However, during rendering, we do not employ the touch-contact sensor, so the initial contact positions and corresponding translation vector should be estimated.

In order to construct the input vector, the initial contact between virtual tool and object surface is required. This indicates that the shape of the object must also be provided to the rendering algorithm. One way is to use 3D mesh model of a tool for collision detection. However, this approach requires perfect reconstruction of the mesh model. Instead, we decided to build a *Collision Model* out of the local initial contacts from the training set. The collision model is a mesh model where its vertices are taken from the unique initial contact points measured for the haptic model. In order to build the mesh model out of arbitrary point set, we utilize a 3D mesh processing software (MeshLab; ISTI-CNR, Pisa, Italy). The main benefit of such design is that the collision model perfectly matches the haptic model that ensures stability in rendering.

### 4.4. Rendering Application

During rendering, the haptic model is used for force response estimation. The polynomial and weight coefficients from Section 4.3 are used for computing force output using Equation (2). The input vector is estimated based on the contact point between the collision model and the object surface. The force feedback was estimated at 1000 Hz rate. In order to incorporate the haptic simulation with visual rendering, we build another mesh models for the visual simulation through a 3D reconstruction software (Remake 2017; Autodesk, Inc., San Rafael, CA, USA) with 150 pictures of each target tool. The deformation of the visual model is not considered (mesh model penetrated the surface), since it is used only for navigation purpose. The visual rendering was running in a separate computing thread in 60 Hz update rate. The visual mesh model was manually aligned with collision and haptic models.

## 5. Numerical Evaluation

In our quantitative evaluation, the main object was to assess the error of the rendered forces in compare to the recorded ground truth forces. In an ideal case, the error in the rendered signal should be perceptually negligible. A person distinguishes the change of the stimuli when the amount of this change exceeds the level of Just Noticeable Difference (JND) of the reference stimuli. The JND level follows the Weber-Fechner law for reference force, δI/I=k, where *k* is the Weber constant, *I* is the reference intensity of the stimuli, and δI is the minimal addition of the intensity required for the change to be perceived. Hover et al. proposed an evaluation framework for haptic rendering algorithms [50]. They considered the force magnitude error as a change δI from the reference stimuli *I* and compare the ratio δI/I with the JND curve. In this study, we decided to evaluate our rendering algorithm in the same manner. A brief review of evolution approaches for some specific rendering application can also be found in [50].

### 5.1. Procedure

In the current experiment, we decided to perform ten-fold cross-validation for the evaluation of the estimated force signals. The raw data of each tool was randomly split into ten subsets. Nine subsets were used for training and one was used for testing. The testing process was repeated ten times where each subset was used for testing once. On each testing iteration, a representative training set of input-output pairs was selected from the whole training data, i.e., data in nine training subsets using the SMASS algorithm introduced in [46]. The threshold value $\tau_{1}$ for the algorithm was selected empirically as suggested. The average number of selected samples is shown in Table 1. In testing, force responses were simulated based on the input vectors from the testing set.

### 5.2. Results and Discussion

**Figure 6:**
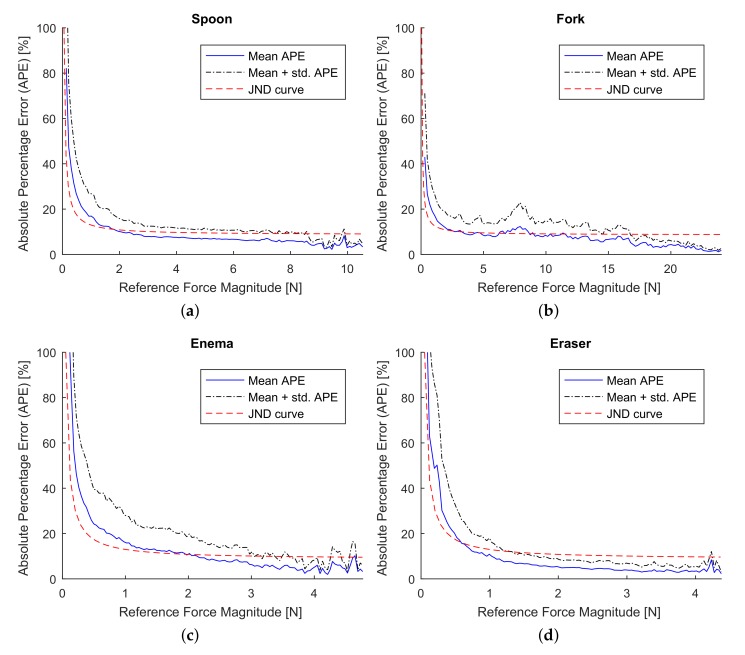
Relative error of the approximated signals for four datasets. The value of the relative error is expressed by absolute percentage error, which denotes the ratio of the force error magnitude to the magnitude of the reference force. The JND curve was computed based on data from psychophysical study in [50]. (**a**) Relative error for Spoon dataset; (**b**) Relative error for Fork dataset; (**c**) Relative error for Enema dataset; (**d**) Relative error for Eraser dataset.

**Figure 7:**
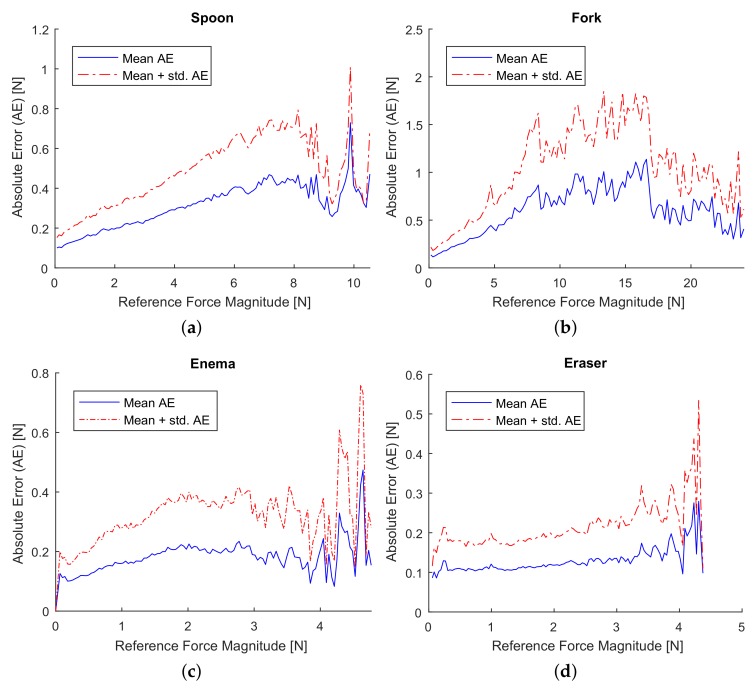
Absolute force error magnitude of approximated signals over the measured force magnitudes for four datasets. (**a**) Absolute error for Spoon dataset; (**b**) Absolute error for Fork dataset; (**c**) Absolute error for Enema dataset; (**d**) Absolute error for Eraser dataset.

Results from numerical experiment are summarized in Figure 6 and Figure 7. The relative error was measured by Absolute Percentage Error (APE), $E_{APE}=100\times ∥\tilde{f_{i}}-f_{i}∥/∥\tilde{f_{i}}∥$, where ∥·∥ denotes the Euclidean norm, $\tilde{f_{i}}$ and $f_{i}$ are measured and approximated forces, respectively. In Figure 6, we plotted the numerical results in form of mean APE over measured force magnitudes. Similarly, the Absolute Error (AE), $E_{AE}=∥\tilde{f_{i}}-f_{i}∥$, is summarized in form of mean AE over measured force magnitudes (see Figure 7).

Numerical evaluation has shown consistent results for all datasets. This indicates the robustness of our approach and gives a room for analysis of general characteristics of the proposed simulator. The MAPE is slightly lower than the JND curve in most cases for all datasets (see Figure 6), and the mean + standard deviation still well coincides with the JND curve. This indicates that the estimation error from the algorithm is perceptual marginally significant. However, the level of the MAPE is barely higher than the level of JND for low reference forces.

One source of the relative force error excess for the low reference forces could be a permanent absolute error due to the low resolution of the gimbal encoder. The angular resolution of the pitch and roll encoders is 0.045°, and 0.18° of the yaw encoder. If a point on the tool’s surface is 10 cm away from the tool’s origin, the maximal position error will be around 0.33 mm. This can be one of the causes of the absolute error for the complete range of forces observed in Figure 7. For instance, if we assume that the tool’s stiffness is around 0.3 N/mm, the position error due to the low resolution of the encoder, which is around 0.33 mm maximum in our case, can induce maximum 0.1 N absolute force error. This absolute force error can affect both the model training and testing phases, resulting in maximum 0.2 N error or on average 0.1 N. The average stiffness of the fork tool was indeed 0.26 N/mm (the average force error due to the low resolution of encoders can approximately reach the level of 0.09 N) and a mean absolute force error for minimal reference forces of the fork data set is approximately 0.1 N (see Figure 7b), which coincide with the above calculation. Thus, we believe that the rate of the relative error can be reduced by replacing encoders to more precise ones.

In case of intermediate reference forces, as it was expected, the absolute error increases linearly, meanwhile the relative error of ‘Spoon’, ‘Enema’, and ‘Eraser’ datasets remains below than JND level. The relative error of the ‘Fork’ dataset is comparable to the JND line with a small spike of the error over the JND line for reference forces around 7 N. Even though the relative error for high reference forces was below than JND line, small fluctuations of the relative error are observed. These fluctuations indicate the lack of data around the high force region where forces are estimated from only a few collected data samples.

## 6. Psychophysical Evaluation

This section evaluates perceptual characteristics of the proposed system. Total ten participants volunteered for the experiment (seven male and three female). They were compensated for their help. Their average age was 27 (19–37 years old). All participants were right-handed. Three subjects had a moderate experience of using haptic devices, meanwhile, the others were naive.

In the experiment, the participants were asked to rate force feedback similarity between virtual and real tools. The set of real tools were taken from the previous experiment (see Figure 5), and corresponding virtual haptic and collision models were also build based on the data used in the previous section.

### 6.1. Apparatus and Conditions

**Figure 8:**
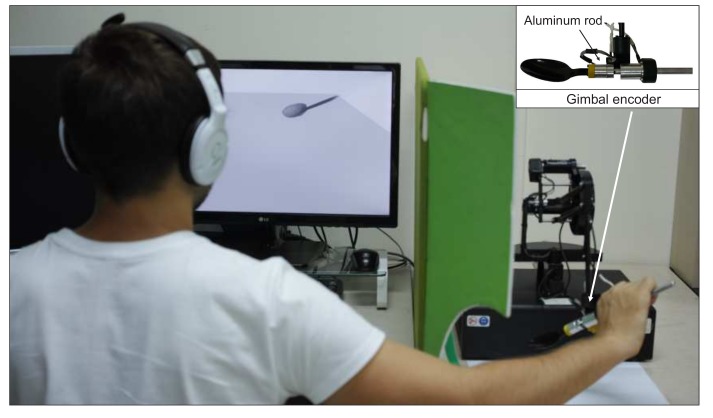
Hardware setup for the experiment. A tool is attached to the gimbal encoder (see top-right) during interaction with a real tool, and detached when the subject interacts with a virtual tool.

The experimental setup of this experiment is depicted in Figure 8. The haptic device is visually isolated from the user by an opaque wall. The gimbal encoder of the haptic device was slightly modified by replacing the force sensor with an aluminum dummy rod that has similar weight and dimension as the sensor in order to minimize the effect of the dynamics of the force sensor in the experimental result (see the inset in Figure 8). A real tool was attached when the user explored the real tool and detached for interaction with the virtual one. The subjects wore headphones playing pink noise to mask their auditory cues.

### 6.2. Procedure

The experiment was performed in two sessions. In the training session, a participant learned how to interact with a virtual tool. Particularly, a subject was instructed to avoid sliding during simulation. The main session was divided into twelve intervals. In each interval, a real tool was first presented to the participant for exploration. Then the real tool was removed, and four different virtual tools were presented in series. He or she was asked to rate the force feedback fidelity of the virtual tools in comparison with the real one. In the subsequent session, the next real tool was attached and presented, and the same comparison was conducted. The presentation order of the virtual tools within an interval and that of real tools throughout the experiment were all randomized. Each participant explored a particular tool three times, and thus there were 48 comparisons per participant in total (48 = 4 × 4 × 3). In each comparison, a participant was asked to rate similarity between real and virtual tools on a scale from 0 to 100 where 0 meant complete discrepancy, and 100 represented exact similarity. The average time of the experiment was approximately 120 min for each participant. Every 30 min of the experiment, the subject was offered to have a five-minute break between intervals.

After the experiment, the participants were interviewed. They were asked to share the overall impression about the proposed simulator and about positive and negative aspects that affected the realism of the rendered signal. Additionally, we asked them to characterize the feedback pattern of each virtual tool and to explain which corresponding real-virtual pair of tools was challenging to match.

In order to build a reference score of the similarity ratings, we conducted an additional psychophysical experiment as a control set-up. The experimental procedure of this experiment resembles the first one. The only difference between them is that in the second experiment the participants compared two real tools and rated similarity. Results from the second experiment can be used as a reference point for the highest similarity score. If similarity rating between a real tool and a virtual tool built from the real tool is comparable to this reference point, it could be confirmed that the perceptual difference between real and virtual tools is at the same level of that between two same real tools. Two psychophysical experiments were conducted on different days.

### 6.3. Results and Discussion

**Figure 9:**
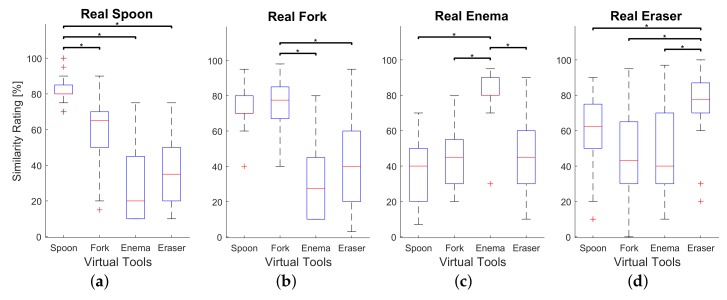
Similarity ratings between each real tool and all virtual tools. Asterisks indicates significant differences between conditions (*p* < 0.05). (**a**) Similarity scores for real Spoon; (**b**) Similarity scores for real Fork; (**c**) Similarity scores for real Enema; (**d**) Similarity scores for real Eraser.

**Figure 10:**
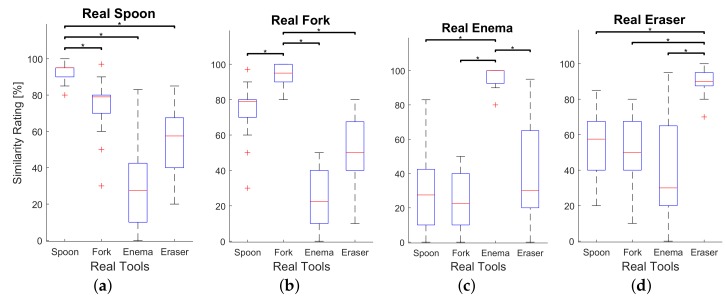
Similarity ratings between each real tools. Asterisks indicates significant differences between conditions (*p* < 0.05). (**a**) Similarity scores for real Spoon; (**b**) Similarity scores for real Fork; (**c**) Similarity scores for real Enema; (**d**) Similarity scores for real Eraser.

The main objective of the psychophysical evaluation is to assess the realism of the force feedback during rendering of the virtual tool deformation. The realism of the rendered forces was measured by means of similarity ratings provided by participants. The similarity ratings for each real-virtual pair of tools are summarized in box plots (see Figure 9) and can be compared to the similarity ratings between real tools, depicted in Figure 10. As it is clearly seen, the median similarity score was rated around 80 percent for all real tools with corresponding virtual analogues, which is close to the comparisons between corresponding pairs of real tools rated approximately 95 percent. Furthermore, the deviation of similarity ratings between corresponding real-virtual pairs of tools is considerably lower than between not matching pairs. A similar trend can be observed in Figure 10. This shows that all participants were consistent in the scoring of highly similar pairs, both in real-virtual and real-real comparisons, which again indirectly confirms that the real-virtual similarity is quite high and consistent.

We performed a two way ANOVA test for both psychophysical experiments in order to compare means of similarity ratings corresponding real-virtual pairs of tools with means of the other pairs for statistically significance. Similarity ratings were considered as dependent variable whereas the subjects and real-virtual pairs of tools were treated as independent variables. Results of the post-hoc test using Bonferroni method are provided in Figure 9 and Figure 10 where asterisks show significant differences between similarity ratings of each matching pair of tools and other combinations of the target real tool with not matching tools. The difference between corresponding pairs of real-virtual ‘Spoon’, ‘Enema’, and ‘Eraser’ and other conditions statistically significant (see Figure 9a,c,d). However, there was no significant difference between similarity scores for the real-virtual pair of the ‘Fork’ and the combination of real ‘Fork’ and virtual ‘Spoon’ (see Figure 9b), which means that participants tend to confuse virtual ‘Spoon’ and real ‘Fork’. Since real ‘Spoon’ and ‘Fork’ are made of a similar material having a common thickness, the stiffness of the global deformation could resemble. The participants might judge the similarity relying upon the global stiffness of the object avoiding other features of the force feedback. Several participants commented during a post-experimental interview that they recognized the real ‘Spoon’ based on the feedback, which helped them to find the matching virtual pair. However, they were not sure when the real ‘Fork’ appeared during the experiment. That is probably why participants did not confuse virtual ‘Fork’ when they compared it to the real ‘Spoon’. A substantial discrepancy of the global stiffness of ‘Enema’ from the rest of tools could also help participants to find its matching virtual pair. In addition, several participants noticed that they felt transition point when the deformation exceeded the certain bending angle of both real and virtual ‘Enema’, which increased the realism.

The participants also reported that they felt stiction when they detached the virtual tool from the contact surface. This sticky effect appeared due to a small amount of undesired sliding during the interaction. Normally, the magnitude of the translation vector goes to zero at the moment when the tool loses a contact with the surface. However, when the sliding happened during the virtual tool deformation, the residual forces occur as the magnitude of the translation vector greater than zero.

## 7. Conclusions and Future Work

In this article, we have designed a new data-driven haptic model for rendering force responses from elastic deformation of anisotropic and inhomogeneous tools. The input space of the model is represented in a local coordinate system of the tool, which makes our approach independent from recording and rendering devices. Due to its data-driven nature, our algorithm allows modeling of complex deformations, such as self and multiple contact collisions. Assuming that there is no sliding during the contact between a virtual tool and flat surface, the algorithm also supports tool deformation modeling while rolling contact.

In order to evaluate the proposed model, we built a proof-of-concept rendering simulator where we followed a three-step process of data-driven haptics paradigm, i.e., data-recording, modeling, and rendering. We built a custom recording setup and collected data from four real objects. The raw data was processed and a representative set of samples was selected using the SMASS algorithm. The collision and haptic models were estimated based on the reduced data and were used for virtual simulation. In order to assess the simulator we performed numerical and psychophysical experiments. The evaluation of the algorithm showed that it can efficiently interpolate the force feedback in accordance with the corresponding input. The participants of the psychophysical experiment rated the proposed simulator as realistic.

In our future work, we will seek to solve assumptions that we made in this paper. First, we will improve the model to interpolate force feedback during sliding contact and from objects with arbitrary shape and material. Furthermore, rendering visco-elastic effect will also be incorporated into the model. Second, we will generalize the model so that it can be used along with the other data-driven techniques for rendering haptic properties, such as friction, and haptic texture. Third, in order to eliminate the need for cutting tools, we will improve the data collection setup. 

## Figures and Tables

**Table 1:** The number of samples that were collected and used in the test. Second and third columns represent the number of palpations and collected samples per each dataset. The last column shows the average number of samples in a subset for the 10-fold cross-validation. The value of $\tau_{1}$ is a threshold used in SMASS algorithm.

Name of a Tool	Number of Contacts	Number of Samples	$\tau_{1}$	Selected Samples
Spoon	197	87,128	0.07	1023
Fork	114	46,751	0.04	1004
Enema	132	69,182	0.04	1062
Eraser	121	51,492	0.035	962
